# Inheritance of Cancer

**Published:** 1883-08

**Authors:** 


INHERITANCE OF CANCER.—In the course of a paper on the Local Origin of Malignant Growths, read in the section of pathology at the last annual meeting of the British Medical Association, Mr. Jonathan Hutchinson observed: "It is needful to say a few words as to the inheritance of cancer in its bearings upon the doctrine of its local origin, since an adverse argument has been founded upon it. It has been urged with much plausibility, that a disease which is capable of inheritance must be a constitutional one. No doubt, to some extent, this is true; but the argument must not be pushed beyond its legitimate scope. The laws of inheritance, as with property, so with disease, concern convection, and not origin or production. The inheritance of a fortune is a very different thing from its acquisition, and gives us no clue as to how that may have been accomplished. The causes of cancer, as we meet with it in practice, may, perhaps, be usefully classed as three; senility of tissue, local irritation, and inheritance. Of these, only the first two can rank as true causes; the latter, although practically of great importance, is only a mode of perpetuation of that which the other two have originated. Senility gives proclivity, local irritation excites, and subsequently hereditary transmission may perpetuate. The facts, as regards chimney-sweeps' cancer, give perhaps the best illustration of what I mean. Before this malady was partially suppressed by act of Parliament, I believe it was commonly noted that when the trade of sweep went, as it often did, in a family, proneness to suffer from soot-warts, and for soot-warts to degenerate into cancer, increased in succesive generations. Grandsons and great-grandsons were attacked at earlier ages, and with much greater frequency, than those who were new to the trade. Here, then, we observe the liability to a form of cancer, produced in the first instance by a local cause, perpetuated and intensified by hereditary transmission. We witness the genesis of cancer, and see the shares taken by local irritation and inheritance, and how entirely secondary the latter is as regards the former. If we ask what it is which is inherited in the transmission of cancer, probably the nearest approach to an answer which can be given, will be to say that it is a peculiarity in cell-structure generally; not germs; not a blood-malady, but a special type of cell organization, permitting, with greater ease than in other persons, the injurious influence of local causes. Even in the sweep, whose forefathers have suffered from soot cancer, the transmitted tendency still waits for the exciting cause, and the disease occurs, not in internal and therefore protected parts, but on the same part as it did in his great-grandfather, and under the direct influence of exactly the same cause. Not that I would for one moment doubt that, in some instances, the inherited proclivity may be so strong, that it does not wait for the help of any exciting causes, but manifests its power in the production of a cancer which may be considered spontaneous. It is probable that in this way we ought to explain almost all cases of cancer occurring in very early life; and it may be the fact that, in a few of these, something more definite than tissue proclivity may be transmitted, possibly even germinal matter, especially in those cases in which the parent was the subject of the malady. Thus, then, although I fully admit that in the examination of our patients we must make large allowance for the influence of inheritance, I wholly deny that we can allow it rank as a true cause of cancer."—*British Medical Journal.*

